# Transcriptomic analysis of liver immune response in Chinese spiny frog (*Quasipaa spinosa*) infected with *Proteus mirabilis*

**DOI:** 10.1515/biol-2022-1003

**Published:** 2024-11-19

**Authors:** Wei Liu, Yu-Hui Tao, Jie Chen, Cheng-Pu Lu, Le Zhang, Zhi-Hua Lin

**Affiliations:** College of Medicine, Lishui University, Lishui, 323000, China; Lishui City Forestry Bureau, Lishui, 323000, China; Jinyun County Forestry Bureau, Lishui, 321400, China; College of Ecology, Lishui University, Lishui, 323000, China

**Keywords:** Chinese spiny frog, immune-related gene, transcriptome analysis, *Proteus mirabilis*, liver

## Abstract

The expansion of Chinese spiny frog (*Quasipaa spinosa*) aquaculture has increased the prevalence and severity of diseases such as “skin rot” disease, which is triggered by harmful bacteria. Previous studies have mainly focused on pathogen identification and vaccine development. However, frog immune responses following pathogenic bacterial infection have hardly been investigated. We thus examined the immune response of Chinese spiny frog to skin rot disease caused by *Proteus mirabilis*. The liver transcriptomes of Chinese spiny frog infected with *P. mirabilis* were sequenced using the MGISEQ-2000 platform. We identified a total of 138,936 unigenes, of which 32.35% were known genes. After infection with *P. mirabilis*, 801 genes showed differential expression, with 507 upregulated and 294 downregulated genes. These differentially expressed genes were enriched in pathways related to cytokine–cytokine receptor interaction, TNF signaling, and toll-like receptor signaling, according to Kyoto Encyclopedia of Genes and Genomes analysis. Following *P. mirabilis* infection, immune genes, including *H2-Aa*, *hamp1*, *LYZ*, *CXCL10*, and *IRAK3*, were significantly upregulated, while *NLRP3*, *ADAM19*, *TYK2*, *FETUB*, and *MSR1* were significantly downregulated. The results provide important information on how the immune system of Chinese spiny frog responds to *P. mirabilis* infection and help understand the development of skin rot in cultured frog species.

## Graphical abstract

**Figure:**
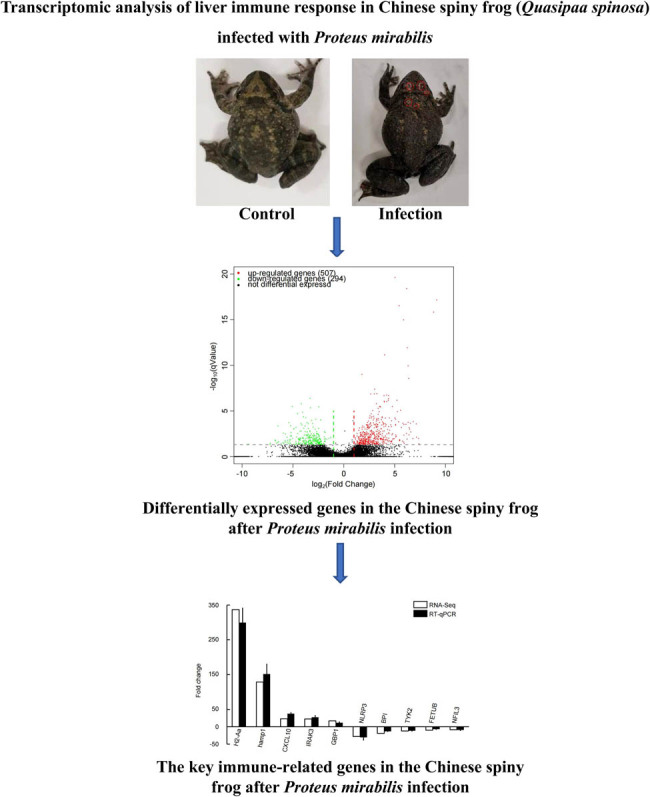

## 1 Introduction

Amphibians are significant components of freshwater ecosystems. However, their habitats are continuously being destroyed by chemicals, resource extraction, and climate change, resulting in declines in biodiversity and populations [1]. The Chinese spiny frog (*Quasipaa spinosa*) is an amphibian species primarily found in southern China. It is of economic significance due to its nutritional value, as evidenced by its muscular tissue containing 18.55–19.39% of protein and being low in fat [2,3]. Chinese spiny frog exhibits several advantageous traits, including a short lifespan, high reproductive rate, and large population size [4]. Although possessing these traits, the population of Chinese spiny frog has decreased by over 30% in the last decade, primarily due to overexploitation, habitat degradation, and fragmentation [5]. Artificial breeding programs have been successful in increasing the numbers of tadpoles and adult frogs since the 1980s.

The expansion of Chinese spiny frog aquaculture has led to an increase in the prevalence and severity of diseases such as “skin rot,” which is triggered by harmful bacteria such as *Proteus mirabilis*, *Pseudomonas fluorescens*, and *Bacillus cereus* [6–8]. Shu et al. pioneered the isolation of *P. mirabilis* from a bullfrog (*Rana catesbiana*) afflicted with severe skin rot disease [9]. The afflicted bullfrogs had various symptoms, primarily skin ulcers on their heads and backs accompanied by white spots, diminished food intake leading to anorexia and empty intestines, thin intestinal walls with mucus accumulation, gas-filled lungs, and enlarged livers [9]. Wang and Xiong subsequently isolated *P. mirabilis* from Chinese spiny frog suffering from skin rot disease and demonstrated the high sensitivity of the involved bacteria to various drugs, including streptomycin, norfloxacin, gentamicin, and kanamycin [8]. Previous studies have mainly focused on pathogen identification and vaccine development. However, there is a significant gap in research investigating frog immune responses following pathogenic bacterial infection. Transcriptome characterization is a rapid and effective method for studying genetics, immunology, and physiology. It allows for the discovery of numerous functional genes [10]. Various aquatic animals and amphibians, such as Neotropical leaf-frog (*Phyllomedusa bahiana*) [11], tiger frog (*Hoplobatrachus rugulosus*) [12], Chinese giant salamander (*Andrias davidianus*) [13], Siberian tree frog (*Rana amurensis*) [14], and dark-spotted frog (*Rana nigromaculata*) [15], have extensively used high-throughput sequencing technology. However, no transcriptome data are available for Chinese spiny frog exposed to pathogens, except for data on the skin transcriptome, which we recently reported [16].

The aim of this study was thus to analyze the liver transcriptomes of infected frogs in comparison to healthy frogs. The goal was to discover novel immune genes associated with skin rot. The results of this study enhance our understanding of the immune response to skin rot in frogs and improve existing databases of amphibian sequences.

## 2 Materials and methods

### 2.1 Sample collection and treatment

Twenty adult male individuals of Chinese spiny frog with an average weight of 150 ± 10 g were purchased from a farm in Lishui City, China. The frogs were separated into two groups and placed in 100 L plastic containers at a water temperature of 18–20°C for a period of 14 days to acclimate them to the laboratory conditions. To induce infection, *P. mirabilis* strain 7570 (ATCC 51286) was grown in a nutrient broth until it reached an OD_600_ of 0.7, as described in a study by Liu et al. [16], and then transferred to a 10 L glass vessel. Frogs were exposed to the culture for 24 h to induce infection, while the control group was kept in nutrient broth without *P. mirabilis*. After confirming that *P. mirabilis* had infected the frogs and caused skin rot disease 24 h after exposure to the culture [16], three frogs in each group were rapidly anaesthetized with 100 mg/mL tricaine methane sulfonate, and their livers were surgically removed, washed in saline, and stored at −80°C. All samples were collected with due permission in compliance with the local license. The methods employed were executed in adherence to the pertinent guidelines and regulations stipulated in the ethics approval and consent to participate section, as well as with the endorsement of the Ethics Committee of Lishui University (Permit No. AREC-LSU202304–043) and ARRIVE guidelines.


**Ethical approval:** The research related to animal use has been complied with all the relevant national regulations and institutional policies for the care and use of animals, and has been approved by the Ethics Committee of Lishui University (Permit No. AREC-LSU202304-043).

### 2.2 RNA-seq

Total RNA was isolated from the livers of Chinese spiny frog-infected and control samples using TRIzol. RNA quantification was performed using a Qubit RNA Assay Kit. Fragmented mRNA containing a polyA tail was isolated and used for reverse transcription, and the resulting cDNA was used to generate double-stranded DNA, which was subsequently phosphorylated at the 5ʹ terminus and ligated with an A at the 3ʹ terminus. PCR amplification products were generated using specific primers and then heat denatured to produce single-stranded DNA, which was then looped with a bridging primer to generate a circular DNA library of single strands. The library was then sequenced using MGISEQ-2000.

### 2.3 Assembly of sequences and annotation of genes

Cutadapt was used to filter the transcriptome sequencing data by removing splicing sequences and low quality reads. Clean reads were then assembled using Trinity (v2.4.0). Assembled transcripts were clustered using TGICL (v2.1) to remove redundancies, resulting in unigenes. Several databases and software, such as CDD, PFAM, SwissProt, KOG, gene ontology (GO), NCBI NR, and NT, were used to annotate the unigenes using NCBI Blast+ (v2.60). Kyoto Encyclopedia of Genes and Genomes (KEGG) annotations were performed using the KEGG Automatic Annotation Server (v2.1).

### 2.4 Differentially expressed genes (DEGs)

Gene expression was measured using Salmon (v0.8.2), and differential expression analysis was performed using DEGseq (v1.26.0) with screening criteria of |FoldChange| >2 and a *Q*-value <0.05. TopGO (v2.24.0) and ClusterProfiler (v3.0.5) were used for GO and KEGG pathway analysis.

### 2.5 RT-qPCR

Validation of the transcriptome sequencing data was performed using qPCR, which involved total RNA extraction with TRIzol, cDNA synthesis using AMV reverse transcriptase, and analysis on a CFX96 real-time PCR system. The qPCR data were analyzed using the 2^−ΔΔCt^ technique [17], with *18S rRNA* as the reference gene. Table 1 lists the primers used in this study.

**Table 1:** Primer sequences used in this study

Gene ID	Gene	Sequence	Amplicon (bp)
DN41651_c1_g5	*H2-Aa*	F: CACACACAGCCTTGACGAAA	181
		R: TCCTGCTGCCTCATATCTGG	
DN42182_c4_g2	*hamp1*	F: GCCCCACAGCAAAACCATTA	158
		R: CGGTGCATCCCTACTGAGAA	
DN38798_c1_g1	*IRAK3*	F: TCCTGGCAAGTACGCTACAA	157
		R: CCATGGCAAAGTCTGAGAGC	
DN42261_c1_g2	*GBP1*	F: AGCGGGTGAAATAGTGGACA	180
		R: TATACGGCAGCACAGACCTC	
DN29732_c0_g1	*CXCL10*	F: CTGACATTTTCCAGCTGCGT	222
		R: ACAAAATGCGTCAGTCCTGC	
DN36031_c1_g1	*NLRP3*	F: TCCCGAGTCATTGAGTGTCC	157
		R: GGGGAAGAGGAGCTGTGATT	
DN28594_c0_g1	*BPI*	F: TGATAGACAAACCCCGCTGT	196
		R: ATTACTCACTGATGGGGCCC	
DN39406_c3_g1	*TYK2*	F: CAGCACAGGAACCACTGAAC	216
		R: TCAGCATTTGTGTCAGTGGC	
DN42797_c2_g2	*FETUB*	F: TCACACCATCACAGAAGGCT	192
		R: AGCTCTGCCTTACCACTCAG	
DN29690_c0_g1	*NFIL3*	F: GGAGTTTGTATTGCGGCGAT	171
		R: AGGTACAGAAGAGCGATGGG	
DN35432_c1_g1	*18S rRNA*	F: CTTTGGTTTCCCGGAAGCTG	246
		R: TGGGGCCATGATTAAGAGGG	

## 3 Results

### 3.1 Sequencing and assembly

A total of 51.89 Gb of data were generated via sequencing using the MGISEQ-2000 platform (Table 2). Valid data were uploaded to the NCBI SRA database under accession number PRJNA1012438. After assembly and elimination of duplicates, a total of 138,936 unigenes were acquired, totaling 88,890,617 bp, with an average length of 639.8 bp and an N_50_ of 964 bp (Table 3).

**Table 2:** Quality control data for samples

Group	Sample	Valid data (Gb)	Q20 (%)	Q30 (%)	GC ratio (%)
Infection	Infection1	10.03	98.23	94.94	47.85
	Infection2	8.93	98.25	95.16	47.10
	Infection3	7.24	98.31	95.31	47.31
Control	Control1	9.59	98.01	94.28	46.53
	Control2	8.36	98.18	94.81	46.61
	Control3	7.74	98.17	94.95	48.44

**Table 3:** Summary of sequencing data using the MGISEQ-2000 platform

	Number	≥500 (bp)	≥1,000 (bp)	N50	N90	Max length (bp)	Min length (bp)	Total length (bp)	Average length (bp)
Transcript	279,582	100,338	50,453	1,186	271	17,310	201	195,977,662	700.97
Unigene	138,936	46,034	20,518	964	260	17,310	201	88,890,617	639.8

### 3.2 Annotation of gene functions

Unigenes were compared using eight databases, with the NT database having the most gene matches (37,021 genes, 26.65%) and the KEGG database having the fewest (8,666 genes, 6.24%; Table 4). GO categories were analyzed, revealing 66 categories in three main groups: biological processes (26 categories, 39.39%), cellular components (21 categories, 31.82%), and molecular functions (19 categories, 28.79%). The annotations of the KEGG database covered 8,666 unigenes in 33 pathways, with a focus on signaling, immune, and endocrine pathways.

**Table 4:** Statistics on the annotation of unigenes

Database	Number of genes	Annotation rate (%)
CDD	13,106	9.43
PFAM	13,191	9.49
KEGG	8,666	6.24
KOG	14,988	10.79
SwissProt	24,837	17.88
GO	16,752	12.06
NR	33,683	24.24
NT	37,021	26.65
Annotated in at least one database	44,947	32.35
Annotated in all databases	2,649	1.91

### 3.3 Screening for DEGs

**Figure 1:**
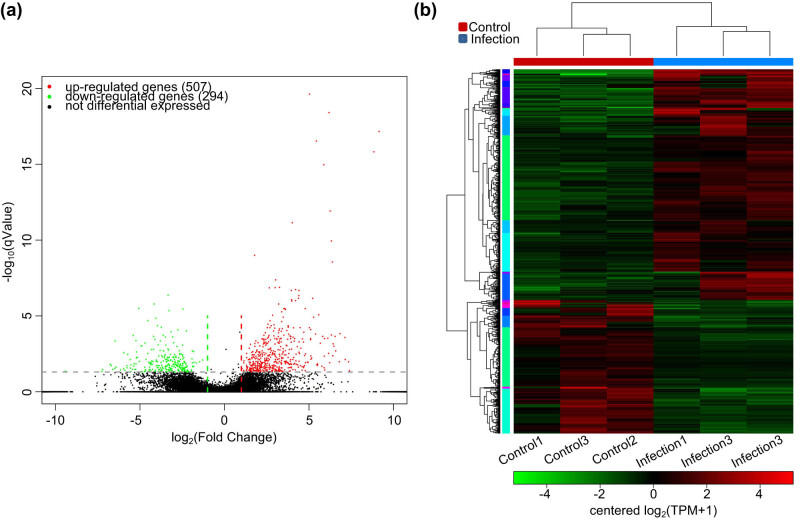
DEGs in the *Proteus mirabilis*-infected and uninfected (control) groups of Chinese spiny frog. (a) Volcano plot of DEGs and (b) clustering thermogram of DEGs.

DESeq analysis identified 801 genes that showed differential expression in the infected and control groups. In the infected group, 507 genes showed increased expression and 294 genes showed decreased expression compared to the control group (Figure 1a). The three biological samples from each of the control and infected groups clustered together (Figure 1b).

**Figure 2:**
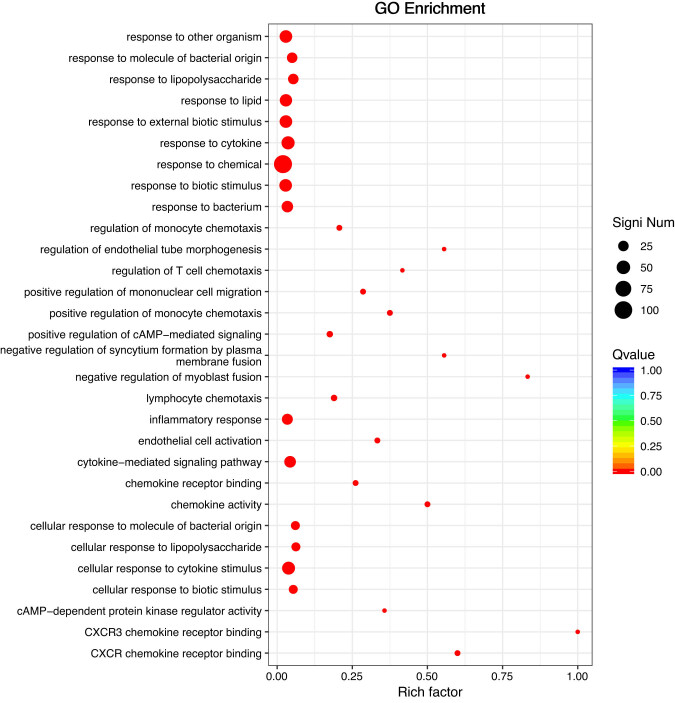
GO enrichment results of DEGs from liver tissues of Chinese spiny frog.

**Figure 3:**
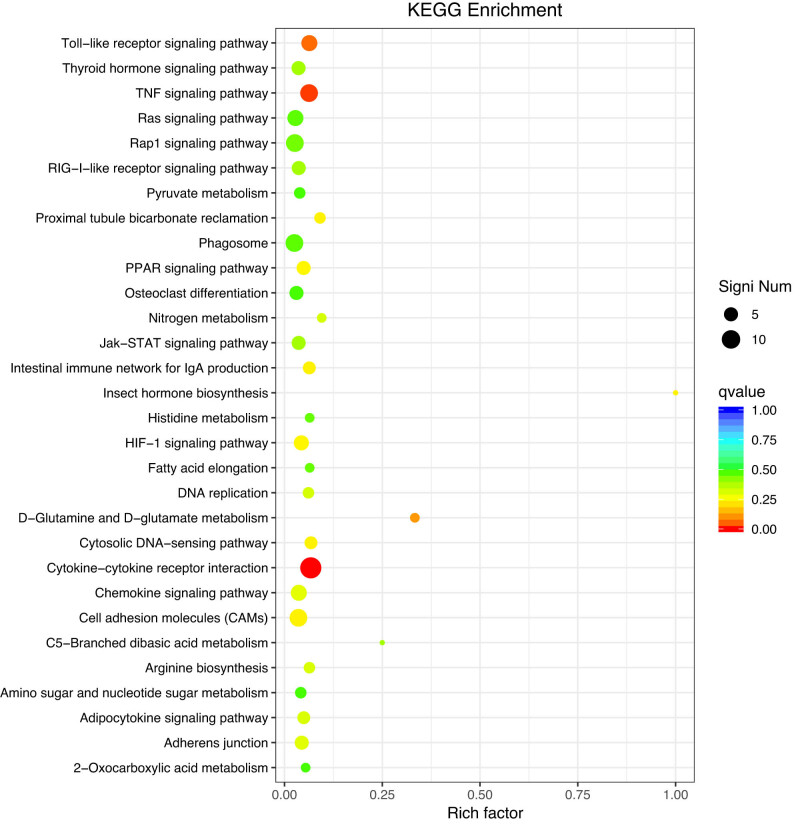
KEGG enrichment results of DEGs from liver tissues of Chinese spiny frog.

Functional annotations were identified by GO enrichment and KEGG pathway analysis of all DEGs. DEGs were enriched in response to chemicals, cytokines, other organisms, external biotic stimuli, and cellular responses to cytokine stimuli in GO classification (Figure 2). KEGG pathway analysis showed enrichment in cytokine–cytokine receptor interactions, the TNF signaling pathway, and the toll-like receptor signaling pathway (Figure 3).

The top 20 immune-related DEGs included ten upregulated and ten downregulated genes (Table 5). In the infected group, the expression of DEGs such as *H2-Aa*, *HA1F*, *hamp1*, *Steap2,* and *LYZ* was significantly higher than in the control group. Conversely, *NLRP3*, *BPI*, *ADAM19*, *CYB5R2*, and *TYK2* showed significantly lower expression levels in the infected group than in the control group.

**Table 5:** Differentially expressed immune-related genes from liver tissues of Chinese spiny frog

Gene ID	NR annotation	log_2_(Foldchange)
DN41651_c1_g5	*H-2 class II histocompatibility antigen, A-Q alpha chain* (*H2-Aa*)	18.35
DN33689_c0_g1	*Class I histocompatibility antigen, F10 alpha chain* (*HA1F*)	14.26
DN42182_c4_g2	*Hepcidin-1* (*hamp1*)	11.34
DN32255_c2_g2	*Metalloreductase STEAP2* (*Steap2*)	6.59
DN43748_c0_g2	*Lysozyme C* (*LYZ*)	5.91
DN30837_c3_g2	*Serpin family E member 1* (*SERPINE1*)	5.44
DN29732_c0_g1	*C-X-C motif chemokine 10* (*CXCL10*)	4.79
DN38798_c1_g1	*Interleukin-1 receptor-associated kinase 3* (*IRAK3*)	4.74
DN42261_c1_g2	*Guanylate-binding protein 1* (*GBP1*)	4.10
DN38730_c1_g1	*TNF receptor associated factor 1* (*TRAF1*)	4.02
DN36031_c1_g1	*NACHT*, *LRR and PYD domains-containing protein 3* (*NLRP3*)	−5.30
DN28594_c0_g1	*Bactericidal permeability-increasing protein* (*BPI*)	−4.37
DN26683_c0_g1	*ADAM metallopeptidase domain 19* (*ADAM19*)	−3.72
DN42088_c2_g1	*NADH-cytochrome b5 reductase 2* (*CYB5R2*)	−3.49
DN39406_c3_g1	*Tyrosine kinase 2* (*TYK2*)	−3.48
DN41812_c0_g1	*Interferon lambda receptor 1* (*IFNLR1*)	−3.43
DN31009_c0_g1	*Syntaxin binding protein 5 like* (*STXBP5L*)	−3.34
DN42797_c2_g2	*Fetuin B* (*FETUB*)	−3.13
DN29690_c0_g1	*Nuclear factor interleukin-3-regulated protein* (*NFIL3)*	−2.92
DN35434_c1_g6	*Macrophage scavenger receptor 1* (*MSR1*)	−2.45

### 3.4 qPCR validation

**Figure 4:**
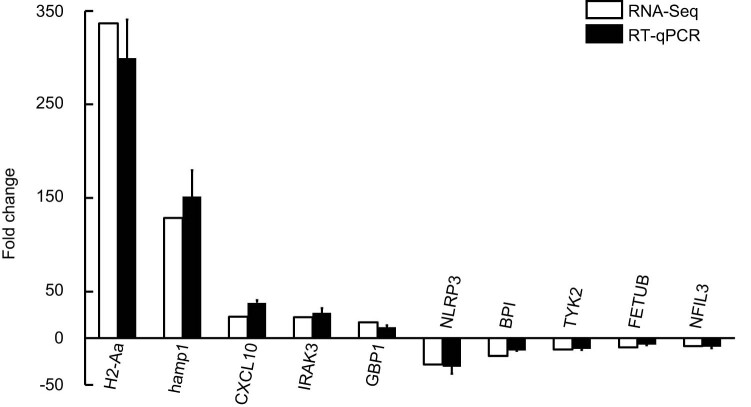
Validation of DEGs from liver tissues of Chinese spiny frog using qPCR. Data are presented as mean ± standard error of the mean for three samples.

To validate the accuracy of the transcriptome sequencing, qPCR was performed on a selection of ten DEGs, specifically *H2-Aa*, *hamp1*, *IRAK3*, *GBP1*, *CXCL10*, *NLRP3*, *BPI*, *TYK2*, *FETUB*, and *NFIL3* (Figure 4). The qPCR results indicated that the differential expression levels of these DEGs were most consistent with the results derived from the transcriptome sequencing analysis. The consistent results provide strong evidence for the reliability of the transcriptome sequencing analysis performed in this research.

## 4 Discussion

The high incidence of skin rot in Chinese spiny frog has led to significant mortality, with significant economic implications for aquaculture production. *P. mirabilis*, the primary pathogen responsible for skin rot disease in Chinese spiny frog, has garnered significant attention from the scientific community [8,9]. *P. mirabilis*, as a conditionally pathogenic bacterium widely existing in natural environments, can cause infections in various animals and humans [18]. Its pathogenic mechanisms are complex and diverse, including direct invasion, toxin release, and induction of immune responses [18]. In animal disease models, *P. mirabilis* has been confirmed to cause damage to the skin and various internal organs [8,9]. Therefore, studying this bacterium in connection with skin rot disease in Chinese spiny frog has important scientific significance and application value.

*P. mirabilis* adheres to the receptors on the surface of skin cells through the action of its surface adhesins, thereby achieving a firm attachment [19]. This attachment not only facilitates the bacterial colonization of the skin surface but also provides an optimal environment for subsequent invasion. After attachment, *P. mirabilis* secretes a series of enzymes and toxins, including hyaluronidase and protease, which can degrade the extracellular matrix and cell walls between skin cells [20]. This allows the bacteria to penetrate the skin barrier and enter deeper tissues. Concurrently, the bacteria elicit a host immune response, resulting in the aggregation and activation of inflammatory cells [18], thereby exacerbating dermal tissue damage. The toxins produced by *P. mirabilis* not only directly damage skin cells but may also cause pathological changes such as localized vasodilation and increased vascular permeability [21], which in turn lead to the appearance of skin redness, exudation, and other symptoms collectively known as skin rot disease.

Despite the increasing problem, there is a lack of studies investigating the possible immune response mechanisms of the host. In this study, we thus used liver tissue of frogs to analyze changes in transcript levels before and after infection with *P. mirabilis* using transcriptome sequencing analysis. Out of 138,936 unigenes, 32.35% were successfully annotated. We thus provide preliminary transcriptome sequencing results of Chinese spiny frog livers, which add to the existing transcriptome database for this species.

DESeq analysis identified 801 unigenes with differential expression levels in the control and infection groups. Five hundred seven unigenes were upregulated in the infection group, while 294 unigenes were downregulated compared to the control group. Numerous DEGs were discovered in key biological processes such as cytokine–cytokine receptor interaction, the TNF signaling pathway, the toll-like receptor signaling pathway, and several other signaling pathways. Toll-like receptors are essential in the immune system for the recognition of PAMPs. When myeloid cells in the innate immune system are activated, toll-like receptors trigger the release of inflammatory cytokines, which then induce lymphocytes to mount an adaptive immune response specifically aimed at eliminating the invading pathogens [22]. For example, changes in the expression of certain inflammatory cytokines may promote the migration of dendritic cells to the skin and induce Th1 or Th17 immune responses [23], thereby worsening the condition of skin rot disease. In addition, liver macrophages may secrete chemokines that attract other immune cells to the skin [24]. Cytokines are essential to the body’s natural defense systems by regulating the immune response and being involved in immune regulation and inflammation. Certain cytokines (such as IL-6 or TNF-α) can reach the skin via the bloodstream and be involved in regulating the activation and function of skin immune cells [25]. For example, studies have shown that IL-6 is significantly elevated in patients with psoriasis and may exacerbate skin inflammation by promoting T-cell activation [26]. After infection with *P. mirabilis*, immune-related genes in Chinese spiny frog livers were mainly concentrated in these three pathways.

Various DEGs related to the immune system were examined, such as *H2-Aa*, which is part of the MHC II complex. MHC II is a crucial molecule involved in the regulation of the immune system, with a significant impact on the presentation of antigens and assisting in the development of T cells. Recent research indicates that unscheduled changes in *H2-Aa* can hinder the production of MHC II and the development of CD4^+^ T cells in mice [27]. In this study, *H2-Aa* expression was significantly induced by *P. mirabilis* infection, suggesting that H2-Aa molecules may be involved in the presentation of *P. mirabilis* antigens to lymphocytes.

Hamp1, a peptide recognized for its ability to fight bacteria, has been shown to be highly effective against a variety of microorganisms [28,29]. Hepcidin transcripts are predominantly expressed in the liver, but can also be detected in various other tissues [29]. Exposure to lipopolysaccharides or poly I:C, together with pathogen invasion, results in a marked increase in hepcidin mRNA levels [30,31]. This study also revealed a significant induction of *hamp1* gene expression following infection with *P. mirabilis*, suggesting a potential role for hamp1 in the immune response to this pathogen.

Lysozymes are crucial immune proteins that play an important role in the initial host defense. The primary mechanism by which lysozymes eliminate bacteria involves the hydrolysis of cell wall peptidoglycan (PG) [32]. In addition, conventional (c-type) lysozymes possess highly cationic properties and can independently eradicate certain bacteria without requiring PG hydrolytic activity [32]. In the ongoing evolutionary arms race between hosts and invading microorganisms, pathogenic bacteria have evolved strategies to resist lysozyme-mediated death [33]. Furthermore, recent research indicates that lysozymes not only act as antimicrobial agents, but also play a role in regulating the host immune response to infection. The degradation of bacteria by lysozymes leads to the release of bacterial products such as PG, which in turn activates pattern recognition receptors in host cells [34]. In the current study, we found that the lysozyme gene was significantly increased when exposed to *P. mirabilis* infection, suggesting that lysozyme molecules may play a role in the clearance of *P. mirabilis*.

CXCL10, a chemokine identified in early research, is produced by several cell types in response to IFN-γ, including leukocytes, epithelial cells, and fibroblasts [35]. Research has shown that mammalian CXCL10 is essential in controlling the movement, growth, cell death, and blood vessel formation of immune cells [36]. In addition, CXCL10 exerts its biological functions by interacting with a specific receptor, CXCR3 [37]. Recent studies have shown that Chinese spiny frog infected with *Staphylococcus aureus* or *Aeromonas hydrophila* has significantly increased the levels of CXCL10 expression in both the spleen and bloodstream. *In vivo*, recombinant CXCL10 shows strong potential to promote the increase of pro-inflammatory cytokines (TNF-α, IL-1β, and IL-8) and splenocyte growth, and induce lymphocyte movement [38]. In this study, we observed that *CXCL10* gene expression was significantly upregulated in response to *P. mirabilis* infection, suggesting the potential involvement of CXCL10 molecules in chemotaxis and activation of immune cells in response to *P. mirabilis* infection.

## 5 Conclusions

We generated a liver transcriptome database for Chinese spiny frog infected with *P. mirabilis* using the MGISEQ-2000 platform. We identified 138,936 unigenes and 801 DEGs following infection. KEGG enrichment analysis revealed that the majority of the DEGs were enriched in cytokine pathways. This discovery offers new insights and methods for the prevention and control of skin rot disease in amphibians, while also providing significant reference for further research into the ecology of amphibian diseases and the mechanisms of pathogen–host interactions. Further studies could investigate the pathogenic mechanisms of *P. mirabilis*, its resistance to antibiotics, and the impact of environmental factors on its transmission. This would contribute to the conservation of rare amphibians such as Chinese spiny frog and the maintenance of biodiversity.
